# Supplementary figures and images for: Integrated analysis of genome-wide association studies and 3D epigenomic characteristics reveal the BMP2 gene regulating loin muscle depth in Yorkshire pigs
Source: PLoS Genet. 2023 Jun 20;19(6):e1010820. doi: 10.1371/journal.pgen.1010820 (PMC10313041; doi:10.1371/journal.pgen.1010820)

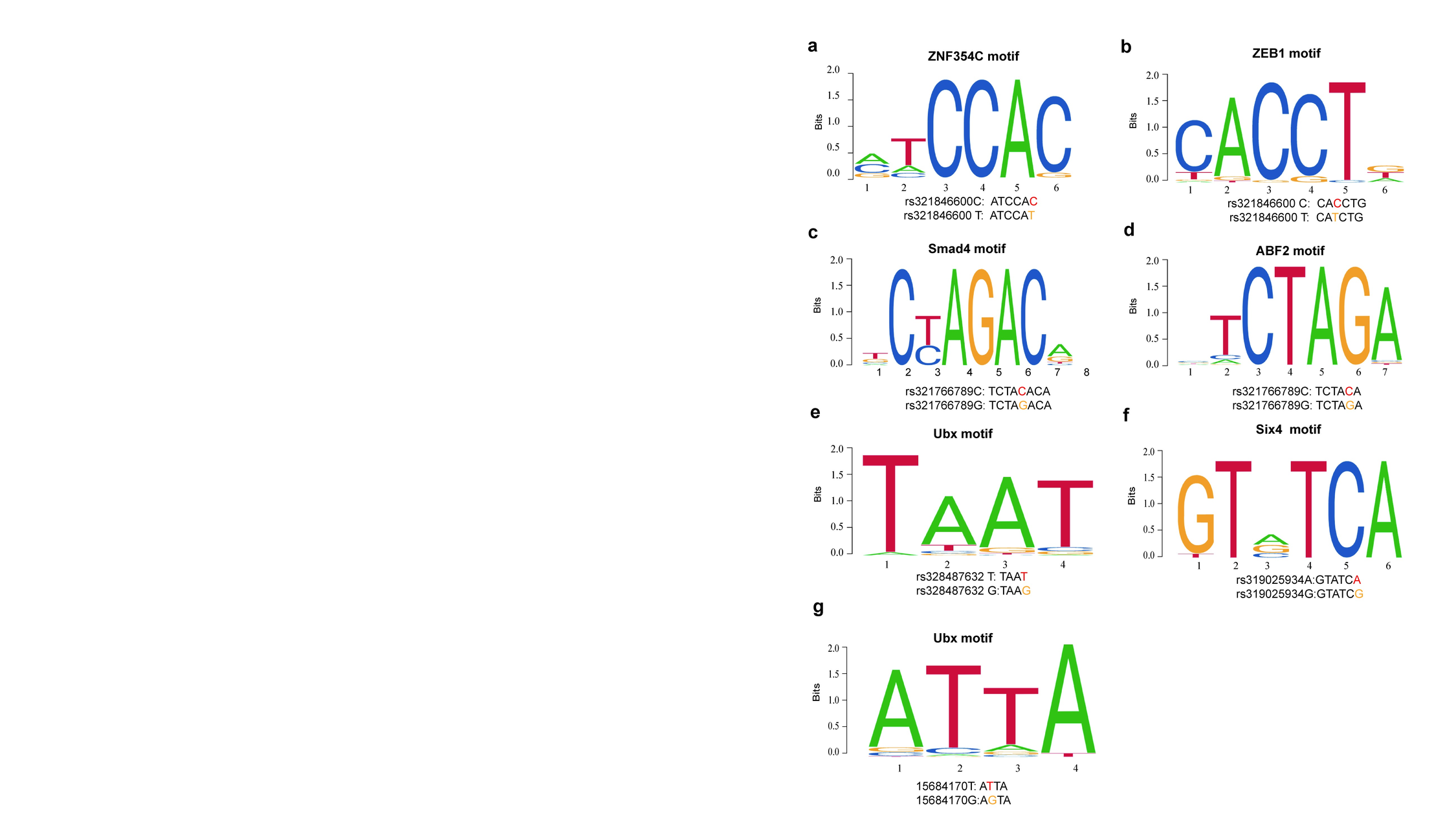

Supplement: S1 Fig — (TIF) [file pgen.1010820.s005.tif]
