# Supplementary material for: Integrated analysis of genome-wide association studies and 3D epigenomic characteristics reveal the BMP2 gene regulating loin muscle depth in Yorkshire pigs
Source: PLoS Genet. 2023 Jun 20;19(6):e1010820. doi: 10.1371/journal.pgen.1010820 (PMC10313041; doi:10.1371/journal.pgen.1010820)
Supplement: Attachment — Submitted filename: reponse letter.pdf [file pgen.1010820.s009.pdf]

Response Letter

Original Submission ID: PGENETICS-D-22-01410R1 (Research Article)

Dear editor and reviewers,

We thank for all the suggestions and comments very much. Based on the review reports, the article has been extensively revised. All of the questions have been answered one by one. The manuscript has been revised carefully according to the suggestions. All the changes in the revised version were highlighted with bright yellow color.

Thanks a lot for all the efforts of the Editor and reviewers.

Tao Xiang, on behalf of all the authors

Reviewer #1:

This paper provides conclusive evidence for a major QTL affecting loin muscle depth (LMD) on chromosome 17 in a population of Yorkshire pigs and suggestive evidence for the functional significance for some SNPs located in putative regulatory regions of BMP2. The problem is that it is unknown if the candidate SNPs affect LMD or not. Therefore, the claim that two SNPs upstream of BMP2 are causative (L 49) is not justified. The authors do not have the genetic resolution to identify causal variants due to the broad QTL region (800 kb) with high LD among markers (Fig. 3). The initial mapping was done with a sparse SNP set, and the resequencing analysis carried out in this analysis show that the most strongly associated markers is located more than 200 kb away from the SNPs now claimed to be causative. The data are worth publishing but the authors need to tone down the arguments that these SNPs are causal it is more correct to state that these are candidate SNPs that may be functionally related to the LMD QTL.

Response： We are very grateful for your thoughtful suggestions. After consideration, we decide to follow your kind suggestion and we modified the statement that "these SNPs are causal" to "these are candidate SNPs that may be functionally related to the LMD QTL " in the paper (Line 49). We agree that due to the lack of direct proof (such as genome editing) that these variants changed the phenotypes, we changed such statement throughout the whole manuscript (such as Line 45-46, Line 369, Line 429-431).

In our regional capture re-sequencing, only SNPs in a narrow region on SSC 17 were genotyped and association analysis for each single SNP was carried out. In the process, it was assumed that all the individuals were independent to each other and no genetic relationships was corrected because we cannot correct such relationships with the limited regional re-sequenced data. This is a limitation for the single-SNP association study. However, when GWAS was performed, p-values of the studied SNPs were calculated when correcting the additive genetic relationships among different individuals, either by using a pedigree-based or genomic-based relationship matrix in the model. Thus, it is reasonable to see different association results in GWAS and the single-SNP association in the target region. This is mainly caused by using different statistical methods.

It is expected that we can see the most strongly associated markers in the re-sequencing analysis is located differently from the associated SNPs in GWAS. The associated markers in the re-sequencing analysis indicate these SNPs' potential importance, but we cannot conclude whether the most strongly associated SNPs are the most vital, because it was based on a not perfect assumption and statistical method.

Actually, the SNPs claimed to be functionally related to the LMD in this study (SNP rs1111440035 and rs321846600 (chr17:15674366)) are located in the most strongly genome-wide association (GWAS) region (15659761 bp to 15755711 bp), which is not more than 200Kb away.

Also, I disagree with the statement that this paper presents a method for fine mapping QTLs. The epigenetic data used here are instructive for identifying functionally important regions of the genome but they cannot be used to exclude sequence variants from being causative and that is what is needed to fine map a QTL. This is the strength of genetic methods like linkage and LD mapping namely that they can exclude regions and thereby narrow down regions associated with a phenotype. What can be done as regards this locus would be to collect similar data from another pig population and also to search for sweep signals in this region. If this is a major locus under strong selection a clear sweep signal is expected among pigs selected for lean meat content. An alternative explanation is that this QTL is due to a haplotype effect caused by multiple closely linked QTLs within this interval so the effect of this region varies among pig populations.

Response: Yes, we admit that "method of fine mapping QTL" is not appropriate, thus we changed the relevant statement and we now only say using epigenetic data can "Narrow down the QTL region that are significantly associated with LMD" (Line 302,303). We don't emphasize that we present a new method now.

We also followed the suggestion that we should collect similar data to search for the sweep signals. We collected 20 American Yorkshire pigs and 24 American Landrace pigs that were raised in the same herd and sequenced them. The sweep signals in 5Mb to 25 Mb on SSC17 were detected, which covered the region that we reported to be associated with LMD, also suggesting this genomic region is vital. We added this to Figure S1.

Specific comments

1.Fig. 3. Please indicate coordinates for the region covered in Fig. 3A in order for readers to compare the regions mentioned in the text. Furthermore, clarify that all SNP data here are based on the sparse SNP chip. This figure illustrates the problem with the identification of causal variants because there is high LD across a large genomic region. The authors focus on the block2 region where BMP2 is located but SNPs in this region show complete or near complete LD with SNPs at the other end of the broad interval about 1 Mb away. Furthermore, it is confusing that the LRT peak is at around 9 Mb on chr 17 according to Fig. 3C whereas the text indicates that the peak is around 15.7 Mb??

Response: Thank you for this insightful suggestion. We have added the coordinates for the region covered in Fig. 3A and the figure legend (Line 744-745).

We agree that the SNPs in the block2 region are nearly in complete LD with SNPs at the other end of the broad interval about 1 Mb away. Note that the LD analysis is based on the "2D" genetic analysis, which cannot illustrate the real spatial distance among different SNPs. According to epigenetic dataset, these "linked" SNPs are located in different TADs or sub-domains. The existing of sequester between different TADs and sub-domains lead to few regulatory relationships between different TADs or sub-domains. Therefore those linked SNPs (e.g.: 1 Mb away) are independent in the transcriptional regulatory function level. The 3D genomic information help to exclude some "linked" SNPs. We added the content in L286-L288.

2. L 298-305, resequencing analysis. The authors focus on the block 2 region 15.65-15.75 Mb, but this resequencing analysis (Table S4) shows that the most significantly associated SNPs are located in the interval 15.89-16.19Mb. The top SNP is located at 16.03 MB more than 280 kb from the candidate SNPs that are functionally evaluated. This is an important result that should be presented in a graph in one of the main figures. Since this is based on a dense SNP data set it provide information about the strength of association across the QTL region.

Response: There are two possible reasons leading to the differences of most significantly associated SNPs between GWAS (>3000 individuals, whole-genome distributed 50K SNP chip) and regional single-SNP association analysis (>700 individuals, a narrow region on SSC 17): (1) When GWAS analysis was performed, P-values of the studied SNPs were calculated when correcting the additive genetic relationships among different individuals, either by using a pedigree-based or genomic-based relationship matrix in the model. However, in our regional capture re-sequencing, only SNPs in the target region were genotyped and association analysis for each single SNP was carried out, which assumed that all the individuals were independent to each other and no genetic relationships was corrected. We cannot correct such relationships with the regional re-sequenced data; (2) the population sizes are quite different. Overall, the differences between the two results were mainly caused by using different statistical methods.

Note that the Single-SNP association analysis cannot be used to conclude which SNP is the "most" significantly associated, since it was based on an imperfect assumption. It is reasonable to see different "peak" SNPs between the two methods. The single-SNP association has some indicative function for SNPs that are significantly associated with the target trait. It was found that there are significantly associated SNPs existing in the block 2 region by using the re-sequencing data, which further confirms the importance of block2 region.

The re-sequencing result was presented in Figure 4c and Figure 4d.

3. L 320. I assume you should cite Fig. 5 here although Fig 5 shows results for only 5 regions and not 6 as indicated in the text. Furthermore, it is not the SNPs that can function as an enhancer, it is regions harboring the SNPs that may have enhancer function. Three constructs are used in Fig 5d: T, G and T-G. The difference between G and T-G is not clear to me and is not explained in the legend. Also, why is this not a candidate SNP when G vs T is significantly different?

Response: We fully agree that the regional harboring SNPs may have enhancer function. We modified the relevant description as "We hypothesized that the genomic regions harboring these five SNPs can regulate the LMD by altering the enhancer function." in L348-349 in the new version. For the SNPs that affect transcription factor binding site, 400bp genome region flanking the significantly associated SNPs were studied. So in total, we should show 6 regions in Fig. 5, which include 6 associated SNPs. However, since the distance between SNP rs328487632 and rs319025934 is 24bp, we only cloned one 800bp genome region that contained these two SNPs. Therefore, we should have 5 regions here.

Note that since the SNP rs319555639 was detected by KASP, but we found out that results of KASP are not reliable (we will explain this point later). Thus, the result of dual-luciferase reporter for SNP rs319555639 was deleted in the revised manuscript. Now in Fig.5, we only have 4 candidate functional genome regions in the new version as follows:

In Fig 5d, we showed four different bars. "15684170T" means for the cloned 800 bp genome that contained the SNP 15684170, at this position, it is the T allele. Similarly, "15684170G" means it is the G allele at this position. "15684170T-G" means we cloned 800 bp genome but mutated the T allele to G allele at this position. We did this because for this 800 bp cloned genome region, it contains more than one SNP. In order to exclude the effects of other SNPs, we have to mutate the allele in position 15684170 to prove it has effect. For the other cloned 800bp genome regions that covered the candidate SNPs, such as Fig.5C (SNP rs321846600), they only have one SNP. We added the information in L359-L365.

4. L 327-335. The description of the promoter polymorphism is a bit confusing. The authors refer to 5 SNPs but Fig. 6 suggests that the correct description is 4 SNPs + an InDel (5bp deletion or insertion), correct? Furthermore, the authors refer to rs numbers in the text but the figures use distance to TSS, these two annotations need to be connected in the text perhaps using the M1 to M5 annotations. Fig. 5e is confusing since constructs indicated below the bar is not annotated and do not seem to have been used so why include them in the figure??

Response: Thank you for your comments. We have changed "5 SNPs" to "5 variants (4 SNPs + 1 InDel)" in our revised manuscript, and we have used M1 to M5 to connect rs numbers in the text and distance to TSS in Fig.6a according to your comments. To clearly display the constructs, we have redrawn Figure 6(ace) in the revised manuscript.

5. Fig 7cd. There is much more comparative data available for mammals that can be used to assess conservation scores for these SNPs. The authors state that both M4 and M5 are evolutionary conserved but that is not correct for M5 because Fig. 7d shows that both C and G occur among other mammals. It would be better to refer these SNPs as REF and non-REF because these are polymorphism. For instance, for M4 the variant denoted MUT is the one present in all other mammals included here.

Response: Thank you for pointing out this problem in our manuscript. We feel really sorry for our carelessness. For M4, C is WT and G is MUT in pigs. We have corrected it in the revised manuscript.

Moreover, GERP scores was calculated from 90 mammals, which is commonly used to measure the conserved nature of gene sequences across species. A high GERP score means that the sequence is highly conserved. Thus, in this version, we added GERP scores for M4 and M5. For M4, the GERP score is 1.91, which indicates it is conserved; for the M5, its GERP score is -5.13, which showed it is less conservative than the M4. We added this content in L392-L397.

6. Gel shift assays, Fig 5f and Fig. 7ef. Firstly, it is not possible to verify causative SNPs with gel shifts, it can merely support their functional significance but it cannot prove causality, change subtitle. The authors should add one more lane to these experiments and test if excess cold probe of the other allele can compete for binding or not. If a mutation inactivates binding, cold mutation probe will not be able to compete with the labeled oligo binding the nuclear factor. For instance, Fig 5f shows that Cold U-WT can compete with B-WT but it is of interest to know if cold U-MUT can compete with B-WT or not. Fig. 7e has a lot of background and is not convincing, it needs to be improved. The authors highlight three bands labeled 1, 2 and 3, they should explain their interpretation which of these are specific and which are not.

Response: Thank you for the above suggestions. We have changed the subtitle to

"Verify important candidate variants by EMSA". We added lane 7 and lane 8 according to your suggestions. Cold U-WT to compete with B-MUT (lane 7) and cold U-MUT to compete with B-WT (lane 8) were added. Results showed that cold U-MUT could not compete with B-WT, which supported that the arrow 1 is specific for WT.

We apologize for this ambiguity. To clarify this issue, we have rewritten this sentence as follows: "For rs1111440035, both the WT and MUT probes detected two common bands (Figure 7e, arrow 2 and 3 in lanes 2 and 5), whereas the WT probe detected an additional shifted band (Figure 7e, arrow 1 in lane 5). When using U-MUT to compete with B-WT, the shift was not eliminated (Figure 7e, arrow 1 in lane 8). Thus, the specific band indicated that some unknown trans-acting factors could uniquely bind to the WT probe and likely respond to the different promoter activities of BMP2 H and BMP2 L." in lines 402-407. The relevant contents are provided in the below Figure 1 for your quick reference.

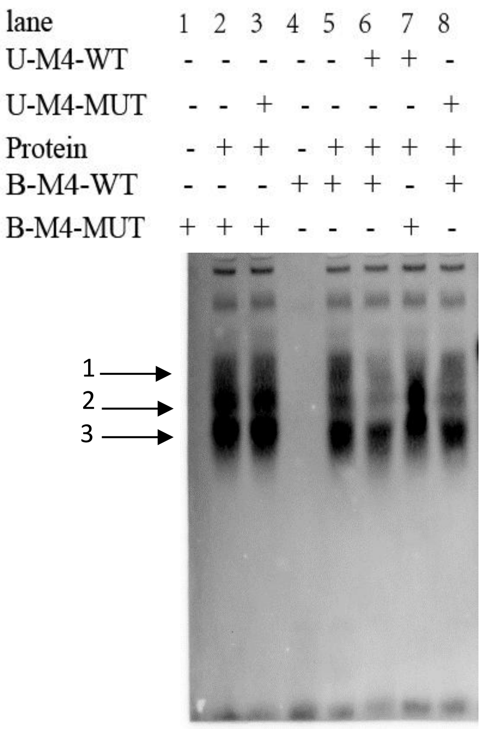

Response Figure 1. The result of EMSA. Here, "protein" represents the PK15 nuclear extracts. U-M4-WT represents the unlabeled M4-WT probe, similar to U-M4-MUT, and B-M4-WT represents the biotin-labeled M4-WT probe, similar to B-M4-MUT.

7. L 367. This is not correct that you fine mapped the QTL you still have a large region, but you provided functional evaluation of candidate SNPs.

Response: We have revised the description at this place and avoid mention fine mapping throughout the paper (Line 282, 302,303,417, 442,486).

8. L. 388-389 I disagree with this statement, it is important to distinguish genetic evidence and functional evidence (see main comment above).

Response: Thanks for your comments. We have revised the description at Line 429-431 as "our method provides conclusive evidence for a major QTL affecting LMD on chromosome 17 and provides candidate SNPs that may be functionally related to the LMD."

Minor comments

1. L 68-70. It is not the number of SNPs that is most relevant here but the number of independent loci associated with the trait, because a single QTL may involve hundreds or thousands of SNPs that show statistical significance if whole genome sequencing has been performed.

Response: We have revised the number of SNPs to the number of independent loci (Line 83-86).

2. L 250. Us the same format consistently for genomic regions, so this should be indicated as 15.08 – 16.76 Mb

Response: Thank you pointing out this error, we have revised the format of genomic regions (Line 285).

3. L 254 I think it is better to write that "the LMD-related causal variants were expected…". This is because you use genetic data to map causal variants but if these are regulatory they may act on distance and the genes regulated by the causal variants maybe located outside the interval.

Response: Thank you for this insightful suggestion. We have changed this part to "the LMD-associated SNPs are closely related to each other, and the LMD-related causative variants and their target genes were expected in this TAD region" in L291-293.

4. L. 265, change "mapping" to "map", one example that some further improvement of English language is needed. Furthermore, should this be "QTLs" in pluralis? I assume you identified a single QTL that may include multiple causal variants but if so, these segregate as a single QTL. (However, the major QTL maybe composed of multiple closely linked QTLs but the authors do not have the genetic resolution to distinguish this architecture from a single QTL scenario).

Response: As recommended by the reviewer, we have enlisted the assistance of an English editor to revise the language. We believe that the updated version has been greatly improved by this revision process. In response to specific comments by the reviewer, the following revisions were listed: (i) for "fine mapping…" was revised to "narrow down." at lines 303 ; (ii) "QTLs" changed to "QTL" at line 303.

5. L 285 Delete "The" at the beginning of the sentence, I assume you have not yet characterized all cis-regulatory elements in the porcine genome.

Response: Done (Line 328).

6. L 293 "two pig subpopulations" please clarify if these are selected from the same Yorkshire population used for the initial QTL mapping.

Response: Yes, "two pig subpopulations" are selected from the same Yorkshire population used for the initial QTL mapping based on the extreme phenotypic values of LMD. We validated the results of KASP and found out the accuracy of KASP is low, thus in the new version, we do not use the KASP results any more. Thank you!

7. L301-305 This sentence is incomplete and the language must be corrected.

Response: We have changed this sentence (Line 333-338).

8. L 396, change to backfat

Response: Done (Line 451,452).

9. L 420-421 It is more appropriate to write binds "an unknown transcription factor".

Response: Thank you for this insightful suggestion. We have modified it (Line 476).

10. Table 2 is sorted by P-value but it is better to sort it based on genomic position since that gives a hint of the peak of association.

Response: We sorted the Table 2 based on genomic position (Line 732).

11. L 686, one decimal place is sufficient here and replace "p-value" with "-log10(p)-value)"

Response: Thank you for your suggestion, we have revised the value to one decimal and replace "p-value" with "-log10(p)-value)" (Line 738).

12. Fig. 2B. Why is this one single TAD-region, there is not much interaction between the 15.08 and 16.76 Mb

Response: We apologize this unclear presentation. The TAD were located at chr17 15080000-16760000, which is 1.68Mb and much larger than the significant SNPs located region chr17 15518001 -16340383. TADs insulate with each other by the boundaries. Thus we just show this single TAD here.

The interaction between the 15.08 and 16.76 Mb was showed in the below Figure 2. We can observe high contact frequency in this region. The resolution of TAD calling using insulation score method is 40 Kb (Response Figure 2c). At this resolution we can observe much more interaction than 10 Kb resolution (Response Figure 2a) but it

hard to observe the detail of the interaction. The Hi-C interaction heatmap at 25 Kb resolution also showed high contact frequency in this region (Response Figure 2b). In order to help the readers to understand the detail we added the resolution information (Res=10kb) at the Fig. 2B and its figure legend.

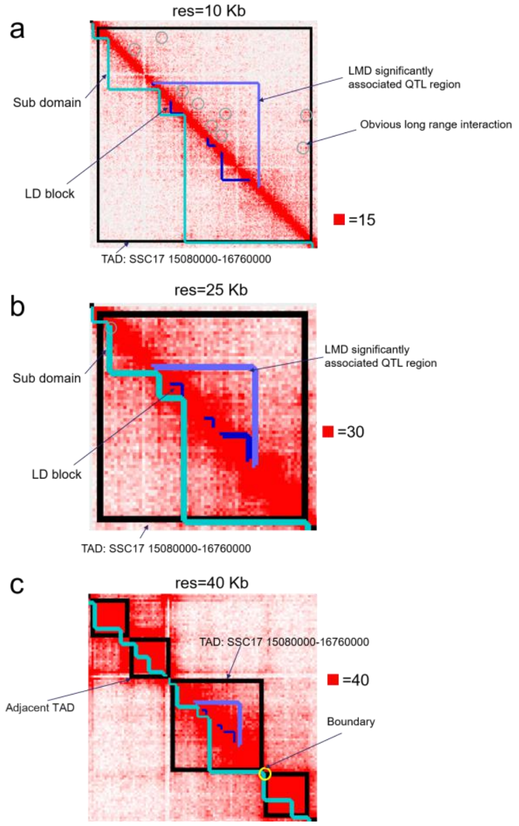

Response Figure 2. The Hi-C interaction heatmap around 15.08 and 16.76 Mb. (a) Interaction heatmap of 15.08 and 16.76 Mb at 10 Kb resolution. (b) Interaction heatmap of 15.08 and 16.76 Mb at 25 Kb resolution. (c) Interaction heatmap of 15.08 and 16.76 Mb and its adjacent TADs at 40 Kb resolution.

Reviewer #2:

Cograts to the authors for the good piece of scientific work showing the have identified not only a major gene, but also a QTN. In my opinion, all the cahnges suggested by the reviewers were accomplished and Engish is in good shape

Response: Thank you for your support of our work.

Reviewer #3:

This paper describes a study in which a very strong statistical association was detected between a region on pig chromosome 17 and a key phenotype of commercial pigs, and subsequently, well-chosen genetic methods were used to dissect the functional differences between SNPs associated with the detected association.

Response: Thank you for your support of our work.

General comments

1. Overall, this is a very interesting paper. The integration of functional analyses with the GWAS and LD-based analyses provide compelling results that implicate specific SNPs from the BMP2 gene in the association with loin muscle depth. My main concern, which could be addressed with rewording in various places, is that the title, abstract and conclusions state that 3D epigenomic characteristics helped them to dissect the basis of the genetic association but I do not see this. As I read the paper, the epigenomic characteristics did not really contribute to detecting the association or narrowing down the candidate SNPs, and rather, the GWAS and LD-based analyses were sufficient to detect the association. What I see as the novelty of the study is the effective use of the dual-luciferase and electrophoretic mobility shift assays to help identify which SNPs drive the strong association detected by GWAS/LDLA.

Response: Thanks a lot for the comments. 3D epigenomic characteristics help us to dissect the basis of the genetic association as follows: (i) we used the TAD around the LMD-associated SNPs to dissect the functions of SNPs. For those SNPs outside the TAD, they have little chance to interact with candidate genomic regions; (ii) with the help of sub-domain data, it shows the candidate genomic region fully covers one sub-domain and the LD block2 is located within the sub-domain. SNPs within the LD block 2 have higher possibility to interact with genomic regions within the sub-domain and thus we narrow down the genomic region to LD block 2; (iii) with the target re-sequence data, we found there were more than 184 SNPs were significantly associated with the LMD. Then we used the epigenetic data (enhancers and promoters) to narrow down the number of significantly associated SNPs. Therefore, we say 3D epigenomic characteristics helped them to dissect the basis of the genetic association. We added more description of the using of 3D epigenomic at line 285-288 and line 309-316.

Another issue in the manuscript is that the results based on target region sequencing are not well integrated with those for the SNP array results. As I understand it, the target region sequencing work was added in response to reviewers' comments on the previous version of the manuscript and these results make an important contribution to the paper. However, the two sets of results need to be combined better to provide a more logical explanation of the study design and results. This applies to the Methods (161-179), Results (285-308) and Discussion (371-389) (some specific issues are also detailed below in Additional comments).

Response: Yes, we added re-sequence data to identify the possible causative SNPs. As we mentioned above, with the target re-sequence data and epigenetic data, we tried to narrow down the number of significantly associated SNPs. In the revised one, we added more description at line 190-196 and at Line 421-426 to better combined results of chip and re-sequencing data. We hope in the new version, it is more clear to show the design of our study.

2. There are some other sections where further details are required (see Additional comments, below). Finally, there are various grammar and wording problems. I have suggested some corrections, but further editing is required in some sections (see Grammar/wording, below).

Response: As recommended by the reviewer, we have enlisted the assistance of PhD-level native English speakers to revise the language throughout the manuscript. We believe that the updated version has been greatly improved by this revision process. In response to specific comments by the reviewer, we have revised the paper according to the revision suggestions.

Additional comments

Methods

1. 107-135: I was confused by the treatment of phenotypes such that polygenic effects have been fitted twice (pre-correction + GWAS), using two different approaches. The authors should explain/justify this further.

Response: We apologize for this confusion. In this part, we first used the SSGBLUP method to obtain the corrected phenotype of LMD, in this step, the LMD of 16533 pigs was recorded and used for phenotypic correction (Line 128). We did this step because SSGBLUP can fit both pedigree and genomic information to accurately estimate the effects. If we directly use those genotyped animals (2733) to estimate the effects, due to the limited number of genotyped animals, it will decrease the accuracy of estimating the effects (Line 143). Then we used the corrected phenotypes of LMD on 2733 genotyped pigs to implement GWAS. Using corrected phenotypes in GWAS is comprehensively used in many other papers.

2. 153-160: The authors should provide further (brief) details here (the reader shouldn't need to read the other paper to understand what has been done).

Response: Thank you for your suggestion, we have added the detailed description at Line 178-185.

3. 163-165: Seven out of how many SNPs?

Response: We apologize for this confusion. In this part, we chosen seven SNPs out of 200 SNPs in 15.65 Mb and 15.75 Mb on SSC17for KASP, and three SNPs were identified significantly associated with LMD.

However, we validated the results of KASP and found out the accuracy of KASP is low. Thus in the new version, we do not use the KASP results any more.

4. 164, 169, 170: Why 15.65-15.75 Mb on line 164, then subsequently 15.51-16.31 Mb?

Response: In this study, we identified a QTL region (15.51-16.31 Mb) significantly associated with LMD. Subsequently, through LDLA analysis, the QTL region 15.65-15.75 Mb was considered being an important candidate QTL region associated with LMD. However, the above-mentioned studies were based on a 50 kb SNP chip. To improve the map resolution and select possible causative variants, we used re-sequencing to genotype the variants between 15.51 and 16.31 Mb. Subsequently the SNP distributed within the significant H3K27ac peaks, which are located within 15.65-15.75 Mb, were chosen for trait association analysis. We described the whole design at the beginning of discussion (Line 414-445).

5. 167: Were the 732 individuals selected from high- and low-LMD groups or across the whole population?

Response: The 732 individuals selected from the whole population. We added the description as "732 individuals, which was randomly selected across the entire population" (Line 189-190).

6. 167-169: Need to provide more details for the target region sequencing.

Response: Thank you for your suggestion, we have added the detailed description for the target region sequencing in Line 190-196.

7. 165, 172…: Which dataset was used for this association analysis described on 174-179? The 393 animals from high- and low-LMD or the 732 individuals?

Response: We apologize for this confusion. In line 165, the association analysis between the SNPs identified by KASP and LMD was implement in 393 animals from high- and low-LMD. In line 172, the association analysis between the SNPs genotyped by the technology of target region sequencing and LMD was implement in

732 randomly selected individuals. As we mentioned above, due to the inaccurate genotyping by KASP, we don't use KASP results in the new version anymore.

8. 180-202: I found this section confusing and further clarification is necessary. It was not clear to me which groups were being compared, i.e. What is meant by "experimental" and "control" groups (202)? Also, which pig(s) were used to generate the cells and provide DNA (189)?

Response: Thank you for your suggestion, we have revised the manuscript extensively. "Experimental" group represents the group was measured following transfection of the vectors with the regions harboring the SNPs that may have enhancer function or promoter function. "Control" group was measured following transfection of empty pGL3-promoter or pGL3-basic vectors. The empty pGL3-promoter or pGL3-basic vector was used as a Control. Dual luciferase reporter assays for different constructs were conducted in PK15 cells. The PK15 cells were bought and genomic DNA was extracted from the ear of Yorkshire pigs. (Line 207-231)

9. 207: As I understand, this was performed for 3 SNPs. Assuming so, this should be stated in the text (e.g. "For the three identified candidate SNPs…")

Response: Thank you for your suggestion. Based on the results of target region sequencing, a total of 5 significantly associated SNPs in regulatory region could affect a transcription factor binding site (Figure S2).Thus, for the five identified candidate SNPs, we implemented dual-luciferase expression assays to verify their functions (Figure 5abcd).

Results

1. 244-246: More details are required regarding the TADs. The reader should not need to read Reference [18] in order to understand this paper. Also see wording comment below.

Response: Thank you for your suggestion, we have added the detailed description and the results for the TADs as "Our previous study identified the boundaries of topologically associated domains (TAD) by Hi-C data of pig muscle tissues. These boundaries showed insulation of interaction between different TADs. The interaction effect of cis-regulatory elements in the same TADs were significantly stronger than that of cis-regulatory elements spanning the two nearest adjacent TADs [18]." (Line 278-281)

2. 284-308: As mentioned above, these results are not integrated clearly with the results from the KASP genotyping. For example, why wasn't there any overlap between the two sets of identified SNPs? More generally, the results of the two approaches should be linked as they are addressing the same aim.

Response: Due to inconsistent sample numbers, there are some differences between KASP data results and targeted sequencing results. In order to verify the consistency between different methods, we compared the consistency between (1) KASP data and re-sequencing data, (2) re-sequencing data and SNP chip data on the same animals. We found the consistency between re-sequencing data and SNP chip data is very high (~99%), but the consistency between re-sequencing and KASP is quite low (~68%). To ensure the results, we decide to discard results related to KASP in the manuscript. Thank you for your precious comments!

3. 353-362: This paragraph needs more details on how to interpret these results, in particular for rs1111440035 or rs80791204. For example, "no additional shifted bands were detected" (359-360), should be following by "suggesting that these alleles did not differ in binding affinity" (if that is correct?). I could not understand the results for rs1111440035 (357-360).

Response: We added more detailed descriptions of the results to the MS. We use lane number to represent groups in the revised manuscript. For example, "the experiment group (lane 2,5)， the competition group (lane 3,6,7,8)".

Yes, we fully agree that "no additional shifted bands were detected" (L359-360), should be following by "suggesting that these alleles did not differ in binding affinity". So, we changed "However, for the WT and MUT probe of rs80791204, no additional shifted bands were detected" to "However ,for the WT and MUT probes of rs80791204, no additional shifted bands were detected (Figure 7f),which suggesting that these alleles are not differ in binding affinity" in the revised manuscript (Line 408-409).

For rs1111440035, both the WT probe and MUT probe detected two common bands (Figure 7e, arrow 2 and 3 in lanes 2 and 5), while the WT probe detected an additional shifted band (Figure 7e, arrow 1 in lane 5). The specific band indicated that some unknown trans-acting factors could uniquely bind to the WT probe and probably respond to the differential promoter activities of BMP2 H and BMP2 L. (Line 398-412)

Discussion

371-389: As mentioned above, this section needs editing to give a more logical explanation of the study design and interpretation of results.

Response: We modified the first and second paragraph in discussion. We use this paragraph to mention all the results and the inner logical of the whole study design. We hope it can help to clarify the paper (Line 414-445).

Grammar/wording

Response: We are very grateful for your thoughtful suggestions to further improve our paper. As recommended by the reviewer, we have enlisted the assistance of PhD-level native English speakers to revise the language throughout the manuscript. We believe that the updated version has been greatly improved by this revision process. We have corrected all the following grammar errors.

76: change "variations" to "variation" (2 places); change "these" to "this"

Response: Done (Line 92)

102: reword, e.g. "SNPs were mapped to the Sscrofa11.1 pig genome assembly."

Response: Done (Line 118)

108: reword, the ssGBLUP analysis incorporates both genetic and non-genetic effects.

Response: Done (Line 125)

150: add "A" before "likelihood ratio test"

Response: Done (Line 172)

170-171: needs rewording (I don't understand what is meant here by "meanwhile distributed")

Response: Done (Line 197)

173: change to "R software" or "R"

Response: Done (Line 201)

180: change to "…promoter and enhancer regions"

Response: Done (Line207)

182: change "predict" to "identify"

Response: Done (Line 209)

183: change to "affect a transcription factor binding site"

Response: Done (Line 210)

185-188: This section needs rewording

--be more precise about their proximity of the two SNPs

--I don't follow why 5 fragments were cloned

--be more precise about "Similar experiments…"

--change "executed" to "carried out"

Response: We apologize for this ambiguity. Since the SNP rs319555639 was detected by KASP, but we found out that results of KASP are not reliable (we have explained this point earlier). Thus, the result of dual-luciferase reporter for SNP rs319555639 was deleted in the revised manuscript. Now in Fig.5, we only have 4 candidate functional genome regions in the new version. To clarify these issues, we have rewritten this sentence as follows: "For the five SNPs in the enhancer region of *BMP2* that affect transcription factor-binding site (Figure S2), we implemented dual-luciferase expression assays to verify their functions. In the enhancer region, 400-bp genome region flanking the significantly associated SNPs were cloned. Since the distance between SNP rs328487632 and rs319025934 is 24bp, we only cloned one 800bp genome region that contained these two SNPs, in total, four fragments in the enhancer region were cloned" in lines 209-214. We have revised "Similar experiments" to "Dual-luciferase expression experiments", and have changed "executed" to "carried out in revised manuscript at line 215.

190: should this be "from genomic DNA"?

Response: Yes (Line 220)

192: change "assay" to "assays"

Response: Done (Line 222)

194: change "cell" to "cells"

Response: Done (Line 224)

217-229: this section requires editing to correct grammar (e.g. verb tenses) and wording

Response: Done (Line 246-259)

234-236: change "standard errors" to "standard error" (3 places)

Response: Done (Line 264, 265, 266)

238: change to "A linear mixed model analysis was applied…"

Response: Done (Line 268)

245: need to reword "insulation of interactions" (I don't have any idea what this means)

Response: We apologize for this issue. we have revised this description in the revised manuscript at Line 278-281.

250: change to "in the same TAD region"

Response: Done (Line 285)

256: add "the" before "above-mentioned"

Response: Done (Line 294)

265-266: change "performed the linkage disequilibrium (LD) analysis" to "investigated linkage disequilibrium (LD) patterns"

Response: Done (Line 304)

274: change "vital" to another word ("compelling"?)

Response: Done (Line 315)

287-288: change to "Based on whole genome sequencing data for 60 Yorkshire pigs"

Response: As we mentioned above, we don't use KASP results in the new version anymore. So this part were delete.

288: remove "the"

Response: As we mentioned above, we don't use KASP results in the new version anymore. So this part were delete.

289: change to "the pig genome"

Response: As we mentioned above, we don't use KASP results in the new version anymore. So this part were delete.

290: should mention this is on SSC17

Response: As we mentioned above, we don't use KASP results in the new version anymore. So this part were delete.

298-308: this section requires editing to correct various grammar and wording problems

Response: Done (Line 331-341)

320: change to “supporting the hypothesis that all 6 of these SNPs can function as enhancers”

Response: Done (Line 355-356)

322: change to “in the enhancer region”

Response: Done (Line 367-368)

323: change “rest” to “remaining”

Response: This part was deleted in new version.

340: change “affecting” to “affect”

Response: Done (Line 382)

344: change “differential” to “different”

Response: Done (Line 387)

357: add “the” before “T>C”

Response: Done (Line 401-402)

359: change “probe” to “probes”

Response: Done (Line 408)

360-362: change to “…this suggests that SNPs rs321846600 and rs1111440035 influence transcription factor binding and affect…”

Response: Done (Line 410-411)

392: remove “the”

Response: Done (Line 448)

392-393: change to “four blocks are found in the LMB-associated region”

Response: Done (Line 449-450)

396: typo (“backfat”)

Response: Done (Line 451,452)

397: do you mean "the most significant SNP"?

Response: Done (Line 453)

398-399: reword sentence; does "this region" refer to that reported in Reference 44?

Response: In reference 44, the significant SNPs associated has been reported within the BMP2 gene, which is belong to the LMD-associated region that we detected. (Line 452-454)

413: change to "This evidence supports the hypothesis that …"

Response: Done (Line 468)

415-427: this section requires editing to correct grammar and improve wording

Response: Done (Line 470-482)
