# Supplementary material for: Integrated analysis of genome-wide association studies and 3D epigenomic characteristics reveal the BMP2 gene regulating loin muscle depth in Yorkshire pigs
Source: PLoS Genet. 2023 Jun 20;19(6):e1010820. doi: 10.1371/journal.pgen.1010820 (PMC10313041; doi:10.1371/journal.pgen.1010820)
Supplement: Attachment — Submitted filename: response Letter.pdf [file pgen.1010820.s010.pdf]

Response Letter

Original Submission ID: PGENETICS-D-22-01410R1 (Research Article)

Dear editor,

We thank for all the suggestions and comments very much. Based on the review reports, the article has been extensively revised. Especially, you mentioned “As reviewer 1 points out, you still oversell your data as providing conclusive evidence that the causal variants are located within a 10 kb region by using epigenetic data. Please tone down this statement.” We are very grateful for your thoughtful suggestions. We have revised the wording of the full paper to realistic describe our results. Moreover, all reviewers’ questions have been answered one by one. The manuscript has been revised carefully according to the suggestions. All the changes in the revised version were highlighted with bright yellow color.

Thanks a lot for all the efforts of the Editor and reviewers.

Tao Xiang, on behalf of all the authors

Reviewer #1:

Reviewer #1: This paper reports convincing evidence for a major QTL on pig chromosome and report functional characterization of candidate SNPs in an enhancer and the promotor of the BMP2 gene. The data are good. However, the remaining problem is that the authors still oversell their data as providing conclusive evidence that the causal variants are located within a 10 kb region by using epigenetic data. The problem here is that the QTL region is large 800 Mb and functional data can neither prove or exclude the importance of a sequence variant for a genotype-phenotype relationship, this can only be achieved by genetic analysis. For instance, a functional assay such as a luciferase assay used here can show that a sequence variant affect function but it does not prove that this effect is important for the genotype-phenotype relationship. Further, functional assay may fail to reveal a significant effect of a sequence variant because the assay does not replicate the conditions when the sequence variant is important. For instance, in this study the authors use a kidney cell line to study the possible effect of sequence variants affecting muscle development (LMD). In conclusion, I think the paper is fine if the authors correct some remaining issues described below and are more realistic what they can and cannot conclude based on these data. Hopefully, my comments below are useful.

Response: Thank you for this insightful suggestion. In this study, we identified a QTL region (15.51-16.31 Mb) significantly associated with LMD. Further LDLA analysis showed the most significant SNP site were located in a LD block, which were named as block2 and was considered being an important candidate QTL region associated with LMD. Moreover, 3D epigenomic characteristics were used to dissect the functions of SNPs. Results showed that the LD block2 is located within a sub-domain and the LD block1, LD block3, LD block4 were outside the sub-domain. SNPs within the LD block2 have higher possibility to interact with genomic regions within the sub-domain, rather than other LD blocks outside the sub-domain. Thus we concluded the LD block2 is an important candidate QTL region for LMD. Further experiments including the dual-luciferase expression assays and EMSA were performed to identify the important variant sites associated with LMD. We agree that we cannot totally exclude the possibility that sequence variants outside this region contribute to the LMD QTL effect because we don't have direct proofs that they do not contribute to the variants of LMD, but according to our results, the LD block2 is indeed an important candidate region for LMD. We have revised the wording of the full paper to realistic describe our results (Line 412-427).

Specific comments

1. L302- (Narrowing down the QTL region). I am still not convinced that these data are sufficient to narrow down the QTL region from about 800 kb to about 10 kb. The reason is that the authors have used a sparse SNP panel. There could be other sequence variants within the 800 kb region with equally strong association to phenotype as those within LD block 2. For instance, Figure 3 shows that the SNP INRA0052824 that is located about 800 kb from the top SNP still shows D'=1 (or very close to 1) to the top SNP in the 10 kb region.

Response: We are very grateful for your thoughtful suggestions. After consideration, we decide to revised "Narrowing down the QTL region" to "Linkage disequilibrium analysis highlights the candidate QTL region harboring BMP2" (Line 288-289).

In this study, we identified a QTL region (15.51-16.31 Mb) significantly associated with LMD and the most significant SNP sites were located in block2. Subsequently, through LDLA analysis, the LD block2 region was considered being an important candidate QTL region associated with LMD. To further integrate the Hi-C data, our results showed the LD block2 independently located in a sub-domain, which indicate the SNPs within the LD block2 have higher possibility to interact with genomic regions within the sub-domain. We agree that we cannot exclude the possibility that sequence variants outside this region contribute to the LMD QTL effect, but our results also suggest the LD block2 is an important candidate region. With the help of 3D epigenetic data, the candidate region was identified.

2. Figure 4d. This figure illustrates very well that it is impossible to identify a 10 kb interval as the sole genomic region harboring causal mutations for the LMD QTL because the strong association with phenotype is over a broad region and may reflect a haplotype effect. To make this figure coherent I suggest that the authors mark their favorite region 15.65-15.75 in Fig. 4d. As far as I can see it is not at all the most strongly associated region in 4d. The authors should also explain in the legend that this plot tests for genetic association to the LMD phenotype.

Response: Thank you for your suggestions. This graph only shows whether such region is important or not, but we do not prefer to emphasize this region in Fig 4d because it does not mean that the SNP with highest p-value is the most important one. In the single-SNP association study, it was assumed that all the individuals were independent to each other and no genetic relationship was corrected because we cannot correct such relationships with the limited regional re-sequenced data, and thereby the results are not conclusive. If mark the region 15.65-15.75 in Fig. 4d, it may erase the importance of the results in GWAS. Fig. 4d just provides clues about important candidate SNPs in the single-SNP association. We followed your suggestion to add details in the legend (Line 740).

3. Figure 5. The text referring to these experiments is a bit confusing. The authors clone 800-bp genomic regions harboring 5 candidate SNPs. The problem

is apparently that these 800 bp fragments may contain other SNPs in addition to the candidate SNP. This becomes apparent in Fig. 5d when the result first is highly significant but then the authors make the specific T to G change in the T construct and then there is no significant difference. The authors conclusion on line 364-365 "that SNP 15684170 is not an important site for enhancer activity" is wrong. This is still possible but the important conclusion is that the highly significant difference between the T and G construct must be caused by another SNP in this interval, perhaps that is a candidate causal sequence variant? The authors end this section by concluding that SNP rs321846600 is the best candidate SNP. However, it is not clear if the 800 bp fragment also carries other SNPs that may be important. The authors need to sequence the four 800 bp fragments and declare which sequence differences each region contains in a Supplementary Table. If the fragment with SNP rs321846600 contains multiple sequence variants they need to mutate the candidate SNP to prove that this is underlying the difference in luciferase activity between constructs.

Response: Among the 5 candidate SNPs, four of them (rs328487632, rs319025934, rs321766789 and 15684170, Fig. 5a, 5b and 5d) were not considered as candidate variants as following:

400-bp genomic region flanking the significantly associated SNPs with different allelic combinations was cloned into pGL3-promoter luciferase reporter vectors respectively. We compared the luciferase activities between different alleles.

For SNPs rs328487632&rs319025934, and SNP rs321766789 (Fig. 5a and 5b), the enhancer activities were not significantly changed between different alleles, No matter how many other SNPs the 800 bp genomic regions contained, these SNPs (rs328487632&rs319025934, and SNP rs321766789) do not play a decisive role.

For the SNP 15684170 (Fig. 5d), we found the enhancer activities are significantly different between 15684170T and 15684170G. However, due to the fact that the 800-bp contains other SNPs, we need to verify if the SNP 15684170 plays the decisive role. Thus, we mutated the T allele to G allele at this position. Results showed that SNP 15684170 is not underlying the difference in luciferase activity between constructs because enhancer activity does not change significantly between the two alleles.

For the SNP rs321846600 (Fig 5c), in the 800-bp genomic region, it only contains this one SNP. Our dual-luciferase expression assays results showed that the C allele of rs321846600 showed significantly higher enhancer activity than the T allele, which implied that the SNP rs321846600 in the enhancer region of BMP2 may play a crucial role in the regulation of BMP2 expression. Furthermore, the EMSA results showed that, for rs321846600, the T>C mutation decreased the DNA–protein binding affinity (Figure 5e). Thus, we considered SNP rs321846600 is the one of candidate SNPs for our study.

This process can be found in Line 332-351.

4. L432-445. I still find the authors arguments here problematic. They are mixing up genetic significance and functional significance. Epigenetic data cannot be used to narrow down a QTL region. It can be used to test the functional significance of SNPs within a QTL region. In this paper the authors have decided to focus on a 10 kb region but they have not excluded that sequence variants outside this 10 kb region but within the 800 kb region contribute to the QTL effect or even is more important than the possible effect of the variants within the 10 kb region. This paragraph needs to be modified to not be misleading to the field. In particular the sentence (The significant SNPs ..) on line 440-441 needs to be followed by something like this: "However, this does not exclude the possibility that sequence variants outside this region contribute to the LMD QTL effect in the genomic region 15.51-16.31 on SSC17."

Response: Thank you for your suggestion, we have revised "dissect the basis of genetic association" to "uncover important candidate functional sites" in Line 415, and modified "the genetic basis of regulating LMD" to "the function of variants governing LMD" in Line 427. In addition, we have added statements that "We admitted that we cannot totally exclude the possibility that variants outside block 2 may contribute to the LMD QTL effects,but the possibilities are much lower than the block 2. " in Line 421-423.

5. L468-471 There is a conflict between these two studies. I recommend that "demonstrated" is replaced with "suggested"

Response: Thank you for your suggestion, we have changed the "demonstrated" to "suggested" (Line 451).

Minor comments

1. L32: delete "the"

Response: Done (Line 32).

2. L34: replace "the key" with "candidate"

Response: Done (Line 34).

3. L45-46: change to "candidate SNPs that may be functionally related to"

Response: Done (Line 45).

4. L51: change "major" to "candidate"

Response: Done (Line 50).

5. L66. Change "loci" to "locus"

Response: Done (Line 65).

6. L69. Change "the major" with "candidate"

Response: Done (Line 68).

7. L277: Change "were" to "are"

Response: Done (Line 266).

8. L346: Since these are predictions, I think this sentence is too strong and should read "Results suggested that these five SNPs may all disturb…"

Response: Thank you for your suggestion, we have revised this part (Line 330).

9. L357 and 359: delete "different carried"

Response: Done (Line 340 and 342).

10. L396: change "supports the fact" with "shows"

Response: Done (Line 377).

11. L397: change "conservative" to "conserved"

Response: Done (Line 377).

12. Figure 5 and 6. Please add a few words to the legend and explain that PK15 is a porcine kidney cell line so that readers don't have to search for this info in the M&M

Response: We have added cell type detail of PK15 in Figure 5 and 6 (Line 746-747 and 761-762).

13. Figure 5e: It is unclear what WT and MUT refer to here. It would be much better to use the nucleotides instead alleles T and C. That would make it much easier to compare the results in Fig. 5c and 5e. It may also be better to change WT and MUT to H and L in Fig. 7 because it is not clear what is WT and MUT given that these are polymorphisms segregating in pig populations.

Response: We apologize for this ambiguity. According to the Reviewer's comments, we have re-drew Fig.5e and Fig.7e-f in the latest revised manuscript as follows:

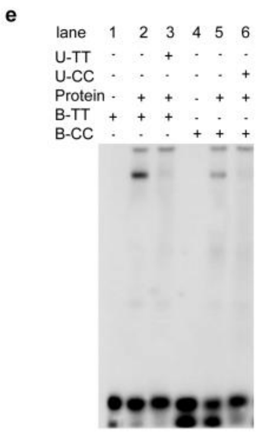

Response Figure 1.EMSA assay for SNP rs321846600. Here, “protein” represents the PK15 nuclear extracts. U-TT represents the unlabeled TT probe, similar to U-CC, and B-TT represents the biotin-labeled TT probe, similar to B-CC.

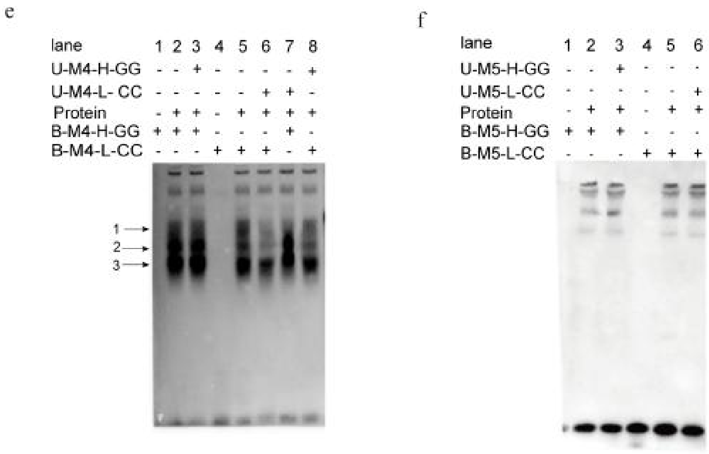

Response Figure 2. EMSA assay for rs1111440035 and rs80791204. Here, “protein” represents the PK15 nuclear extracts. U-M4-L-CC represents the unlabeled M4-L-CC probe, similar to U-M4-H-GG, U-M5-L-CC, and U-M5-H-GG, and B-M4-L-CC represents the biotin-labeled M4-L-CC probe, similar to B-M4-H-GG, B-M5-L-CC, and B-M5-H-GG.

14. Line 415: Add “using a 50k SNP chip” after “pigs” in order to explain that a sparse SNP panel has been used at this step.

Response: Done (Line 395).

Reviewer #3:

Reviewer #3: The authors have made substantial changes to the manuscript and addressed most of my concerns from the previous review. I think it will be suitable for publication following minor changes and editing, as described below. The authors state that they have had assistance from professional editors and/or native English speakers, however, the manuscript would benefit from further editing. There are many places where the language/grammar is problematic.

Response: We thank a lot for the reviewer's supporting of our work. As recommended by the reviewer, we further modified the language throughout the manuscript. We believe that the updated version has been greatly improved by this revision process.

Specific comments

I am not convinced that including F_ST between American Yorkshire and American Landrace breeds is a good addition to the paper. In my view, this weakens what is otherwise a rigorous study. There is no reason to expect that these two breeds will be differentiated in this region of the genome, which has been identified using GWAS within a population of Yorkshire pigs. Or at least, the authors do not provide any justification for this analysis. In terms of the Results, they show a small peak overlapping the region identified by the GWAS but this may be coincidental. There is a higher peak in the 3' direction (about which I also wouldn't interpret too much). As they only looked at F_ST in a small part of the genome, there is no way to tell how extreme these peaks are on the genome-wide scale. I would recommend removing this section of the paper.

Response: Thank you for your suggestion, we have deleted the part.

Lines 278-293: I appreciate the addition of details from the previous study to which the authors refer. However, I found this section somewhat hard to follow. Are the authors saying that the Hi-C data helped them narrow down the region identified by GWAS (line 282)? I don't see how this is the case if the TAD region is actually larger than the GWAS region.

Response: We apologized for the unclear description. As a typical example, a human obesity associated study showed the SNPs in the intron region of FTO gene, actually targeted the downstream IRX3 and IRX5 genes (see below figure). Topologically associating domains (TADs) exhibit higher preference for internal interactions when compared with interactions with the adjacent domains. Yes, in our study, all the candidate sites found are located in one TAD region and the TAD region is larger than the GWAS region. The primary function of the TAD region is to ensure that these loci in the GWAS region do not interact with genomic regions outside the

TAD. Furthermore, with the help of sub-domain data, it showed the LD block2 is located within one sub-domain, which was insulated with other LD blocks. On bias of that, we considered LD block 2 as an important candidate genomic region for LMD. Therefore, we stated that "the Hi-C data helped them narrow down the region identified by GWAS"

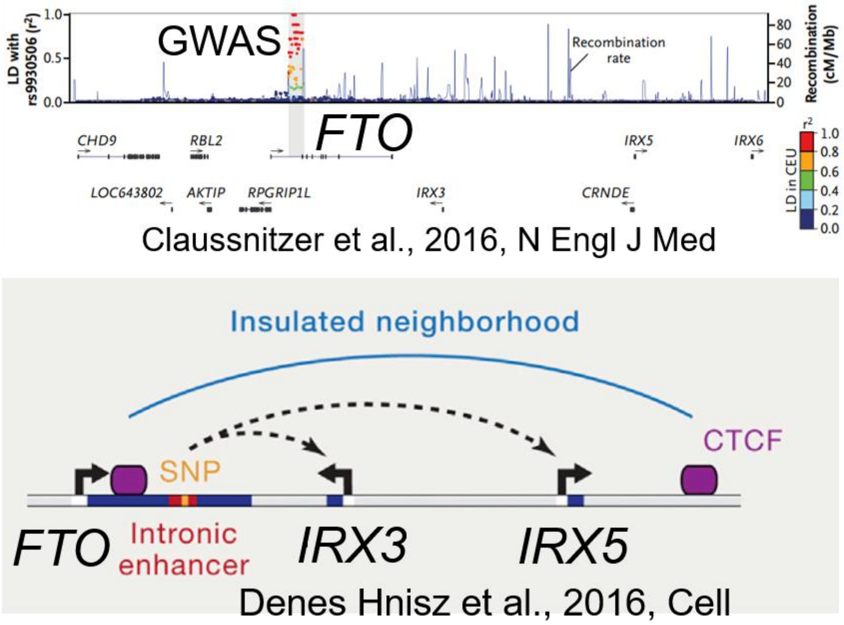

Response figure 1. The illustration of human obesity associated study [1, 2].

--line 279: change "isolate" to another word ("limit"?)

Response: We have revised the "isolate" to "limit" (Line 268).

--need to define "sequesters"

Response: We apologized the unclear expression using the word "sequesters", which actually means "boundaries". We have changed this sentence as "These results demonstrated that the significant SNP-located region had limited opportunity to interact with genome regions located in other TADs due to the presence of boundaries between different TADs." at line 278-280 in the revised manuscript.

--line 286 (and elsewhere in the paper): need to explain in the text what is meant by "interact" and "interactions" in this context

Response: We have added the detail description in this context (Line 269, Line 278-280).

--lines 291-293: please reword to clarify what is meant by "… SNPs are closely related to each other" and "… their target genes were expected in this TAD region"

Response: Thank you for your suggestion. We have revised this part (Line 271-280).

Figure 4d. The legend for this plot needs to be changed to explain that this shows results from an association analysis (as I understand it, as described on lines 199-206?).

Response: Done (Line 740).

Line 348-365: Change "carried" to a different word ("cloned"?)

Response: Done (Line 334).

Line 389: Change "NR2C1 cannot binding" (I don't know what this means)

Response: We have revised the "NR2C1 cannot binding" to "disturbed the binding of the transcription factor NR2C1" (Line 370-371).

Line 397: Change "conservative" to "conserved"

Response: Done (Line 377).

Line 409: Change to either "do not differ" or "are not different"

Response: Done (Line 389-390).

Line 422: Remove "newly developed"

Response: Done (Line 402).

Line 426: Change "verify" to "evaluate"

Response: Done (Line 406).

Line 430: Change "provides" to "identifies"

Response: Done (Line 410).

Line 472: Change to "…increased BMP2 enhancer activity…"

Response: Done (Line 453).

Line 478: Should this be "have previously been reported"?

Response: Done (Line 458-459).

Reference

1. Hnisz D, Day DS, Young RA. Insulated Neighborhoods: Structural and Functional Units of Mammalian Gene Control. Cell. 2016;167(5):1188-200.
2. Claussnitzer M, Hui CC, Kellis M. FTO Obesity Variant and Adipocyte Browning in Humans. The New England journal of medicine. 2016;374(2):192-3.
