# Supplementary material for: Integrated analysis of genome-wide association studies and 3D epigenomic characteristics reveal the BMP2 gene regulating loin muscle depth in Yorkshire pigs
Source: PLoS Genet. 2023 Jun 20;19(6):e1010820. doi: 10.1371/journal.pgen.1010820 (PMC10313041; doi:10.1371/journal.pgen.1010820)
Supplement: Attachment — Submitted filename: reponse letter.docx [file pgen.1010820.s008.docx]

**Response Letter**

Original Submission ID: PGENETICS-D-21-01231	(Research Article)

Dear editor and reviewers,

We thank for all the suggestions and comments very much. Based on the review reports, the article has been extensively revised. All of the questions have been answered one by one. The manuscript has been revised carefully according to the suggestions. All the changes in the revised version were highlighted with bright yellow color.

Thanks a lot for all the efforts of the Editor and reviewers.

Yuanxin Miao, on behalf of all the authors

Reviewer #1:

This paper confirms BMP2 as a candidate gene for loin muscle area in pigs. The authors use a GWAS based on >2,000 pigs and provide evidence for the location of a QTL for loin muscle area in a 800 kb region including the BMP2 gene and a few other genes. Then they combine linkage analysis and linkage disequilibrium analysis and identifies a 10 kb region as the best candidate region for harboring causal variant(s). Within this region they identify a few candidate SNPs that may affect BMP2 expression. The weakness of this paper is that the genetic data is based on a 50 kb SNP chip, i.e., on average about one marker per 60 kb, and therefore the identification of a 10 kb region as the sole candidate region is not convincing. Markers in this region show complete LD also with markers outside block 2 according to Fig. 3, and the poor map resolution does not exclude the possibility that there are other regions within the 800 kb region that show as strong or stronger association with loin muscle area. Therefore the advance this paper do compared with previous reports concerning BMP2 as a candidate gene for loin muscle area in pigs is limited.

  -AU: To further explore the key region related to loin muscle area in pigs, we further used a new technology of target regional sequencing to sequence the associated genome region to increase the reliability of this conclusion. Deeply sequenced (>20X) was performed in the associated region (between 15.51 Mb and 16.31 Mb on SSC17) in 732 randomly selected pigs in the same population. 4980 SNPs were captured in the region and association analysis showed that there were 184 SNPs being significantly associated with LMD (Table S4). To screen the causative variants within the 800 kb region, the epigenomics datasets of pig were integrated. The SNPs that are significantly associated with LMD, located in the cis-regulatory elements and with enhancer-gene correlation were considered as candidate LMD causative variants. To identify the causative variants, we performed both dual-luciferase expression assays and EMSA, and finally two SNPs (SNP rs321846600 located in the enhancer region, and SNP rs1111440035 located in the promoter region) were identified as causative variants affecting the trait of LMD in Yorkshire pigs. The whole paper was largely revised according to the mentioned contents and we hope it make the results more reliable.

Minor comments

  Line 44. Change to ”the major gene regulating variation in LMD”. (There could be other genes that are more important, like IGF2, but they are fixed or close to fixation and do not explain variation in LMD.)

  -AU: Thank you for suggestion, we have change to “the major gene regulating variation in LMD” (Line 47).

Line 55. I disagree with the statement that phenotypic selection is inefficient. It has been very efficient and increased food production tremendously.

  AU: Thank you for suggestion. We have revised this part (Line 60).

Line 60. It is misleading to find citations to studies in humans and cattle after discussing pig traits, these citations should be placed on the previous line after complex traits.

  AU: Thank you for suggestion, We have revised this part (Line 64).

Table 1. Please add info whether loin muscle depth is measured in mm or cm. Text says mm but is that really correct, I assume it should be cm??

  AU: Thank you for suggestion, We have revised this part, we have add the unit of LMD (cm) in the table (Line 669).

Fig. 3a. It is confusing that the markers in this table indicates that this corresponds to the 17-18Mb region and not 15-16 Mb region as indicated in other figures, I assume this simply reflects that these has been named on a previous assembly. This may be explained in the legend, or rename the markers based on the current assembly and include a supp table explaining how markers were renamed. At present the naming is confusing for the reader.

      Further, figure legend for Fig. 3 states that SNPs in red boxes are associated with LMD, but only one SNP is in a red box? Also, it is not clear which the top six LRT points are.

  AU: The reason for this difference is that the chip uses version 10.2, while the analysis of other data is based on version 11.1. Since the chip is named in accordance with the 10.2 version, the figure used the 10.2 version name to draw. The location has been converted to version 11.1 in subsequent analyses. This part was explained in the legend (Figure 3a) (Line 694-696).

The SNP marked in the red box in Figure 3 is the highest LRT value, and the 6 LRT points are marked in the red area in Figure 3c, the detail information of the LRT value was list in Table S3.

Line 555. Reference missing

  AU: Thank you for suggestion. We have revised this part, the missing reference was add in the figure legend (Line 706,708).

Reviewer #2: In this study GWAS and epigenomics data, including ChIP-seq data and Hi-C data, were integrated to identify QTL associated with loin muscle depth (LMD). BMP2 was identified as the most likely candidate gene, and based on the integrated results three SNPs significantly associated with LMD were identified with one SNP located in the promoter region of BMP2 and the other two in the enhancer region. The results are based on state of the art statistical procedures and thus, appears to be solid. I have suggested minor changes in the uploaded version of the manuscript. However, it still needs to be corrected by an expert in English.

 AU: Thank you very much for your positive encouragement. We further added experiments to make the results be more solid. We have invited an expert in English to revise the paper. If necessary, we will ask a native English speaker to further improve the paper.
